# Understanding Colorectal Cancer Patient Experiences with Family Practitioners in Canada

**DOI:** 10.3390/curroncol31060237

**Published:** 2024-05-30

**Authors:** Patil Mksyartinian, Neha Mohammad, Petra Wildgoose, Barry D. Stein

**Affiliations:** 1 Colorectal Cancer Canada, Westmount, QC H3Z 2P9, Canada; patilm@colorectalcancercanada.com (P.M.); neha.mohammad@mail.mcgill.ca (N.M.); 2 Sunnybrook Health Sciences Centre, Toronto, ON M4N 3M5, Canada; petra.wildgoose@sunnybrook.ca

**Keywords:** colorectal cancer (CRC), family practitioner, early-age onset, primary care provider, misdiagnosis, self-advocacy, CRC symptoms, CRC awareness, patient-centred care

## Abstract

Despite ongoing screening efforts, colorectal cancer (CRC) remains a leading cause of death in Canada. The aim of this study was to better understand the experiences of Canadian CRC patients with their family practitioners (FPs) during and after their CRC diagnosis. Patient-reported data were collected through an online questionnaire to understand their CRC diagnosis experiences and identify potential gaps in care. Various factors contributing to challenges throughout a patient’s CRC diagnosis (e.g., delayed CRC diagnosis) were determined using descriptive, qualitative, and inferential analyses. These factors could be targeted to optimize CRC care. This study found that 40.6% of the 175 respondents were unaware of at least one of the following aspects of CRC prior to their diagnosis: early-age onset (EAO), symptoms, and screening procedures. While 84.6% had access to a family physician (FP) before their diagnosis, only 17.7% were diagnosed by FPs. Higher proportions of younger individuals experienced misdiagnoses and felt dismissed compared to older individuals. Only half felt fully informed about their diagnosis when it was explained to them by their FP, while 53.1% had their diagnosis explained in plain language. Transitioning towards patient-centred care would promote pre-diagnosis CRC awareness, address differences in management of CRC care (e.g., dismissal and support), and accommodate for age and health-literacy-related disparities, thereby improving CRC care pathways for patients. Future research should investigate FPs experiences in detecting CRC cases to develop educational resources and recommendations, enhancing early detection and improving patient outcomes (1).

## 1. Introduction

In Canada, colorectal cancer (CRC) is the second leading cause of cancer-related death and the fourth most common cancer type in adults [1]. An estimated 1 in 16 Canadian men and 1 in 18 Canadian women will develop CRC during their lifetime, and an estimated 1 in 38 Canadian men and 1 in 43 Canadian women will die of CRC [1]. Individuals who are diagnosed at the earliest stage (Stage 1) have the greatest chance of five-year survival, at 92% for colon cancer and 91% for rectal cancer [2]. 

The Canadian Task Force on Preventive Health Care (CTFPHC) has highlighted that CRC screening can reduce the incidence of late-stage colorectal cancer and colorectal cancer mortality as demonstrated in randomized controlled trials (RCTs) [3]. According to the CTFPHC guidelines, average-risk adults aged 50 to 74 years should get screened for CRC every two years with a FOBT (FIT or gFOBT) or flexible sigmoidoscopy every 10 years [3]. While significant resources have been dedicated to increasing CRC screening in Canada with some success, gaps throughout the diagnosis pathway remain. Beyond CRC screening, it is significant to understand the enablers and barriers throughout a patient’s diagnosis pathway. The diagnostic process begins when a patient first seeks medical care for their symptoms and ends when a correct diagnosis is given for these symptoms [4]. Along the diagnostic pathway, some factors can impact a patient’s ability to receive a timely diagnosis, such as lack of awareness of CRC symptoms, dismissal of symptoms, and misdiagnosis. A study analyzing associations between experiencing the “alarm symptoms” of CRC and accessing a family practitioner (FP) revealed that fear, embarrassment, and worry about wasting the FPs time are barriers to seeking appropriate CRC care [5]. 

The diagnostic process is complex and requires a physician to take a patient-centred approach that involves information gathering and clinical reasoning to determine a patient’s health problem [6]. Patients’ self-efficacy, such as their ability to prioritize their health and seek appropriate care, is impacted by patient-centred approaches [7]. Particularly, patient-centred communication has been found to improve the quality of care, self-efficacy, and trust in doctors amongst cancer patients [6]. The FP must not only clinically assess patients and provide appropriate care but ensure patients are aware of their options and engaged in their own care [7,8]. 

Although multiple studies exploring patient perspectives exist, few focus on the perspectives of Canadian CRC patients and the critical role FPs play in ensuring that patients have access to appropriate care throughout the diagnostic process [5,7,8,9,10,11]. To improve the diagnostic process, the views of both patients and FPs need to be explored to identify gaps and opportunities on both sides that can be targeted to optimize CRC patient outcomes [7,8]. The aim of this study was to identify the experiences that Canadian patients had with their FPs before, during, and after their CRC diagnosis. The primary endpoints included FP accessibility and dismissal/misdiagnosis of CRC. The secondary endpoint was CRC awareness prior to CRC diagnosis.

## 2. Materials and Methods

### 2.1. Study Design

**Figure 1:**
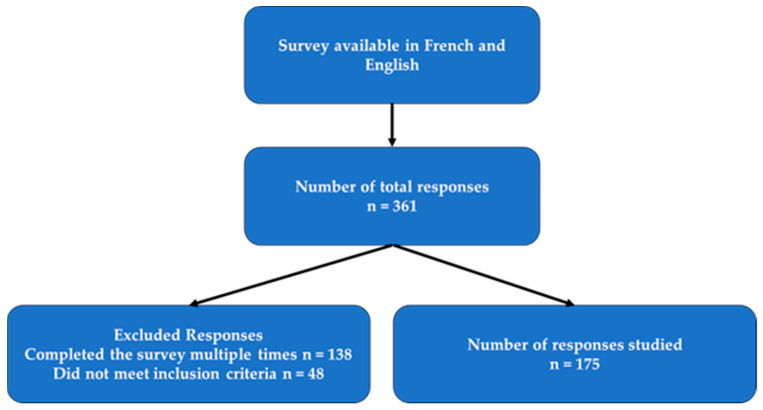
Recruitment and selection of responses.

A cross-sectional study was conducted using an online questionnaire. An online survey platform, Qualtrics, was used to create an online survey (Figure 1). The questionnaire contained four sections and 59 questions (including 1 open-ended question) comprising standardized and author-developed questions. The Colorectal Cancer Canada (CCC) team and an expert advisory panel (EAP) of family physicians reviewed the English and French versions of the questionnaire to ensure the medical accuracy and applicability of the survey within the Canadian context. The questionnaire was available in both English and French. Answering the mandatory consent question granted participants access to participate in the online survey. The four survey sections included in the survey were as follows: Patient Demographics, CRC Awareness, Pre-CRC Diagnosis Experience, and Eventual and Post-CRC Diagnosis Experience. The Pre-CRC Diagnosis Experience section contained five subsections Symptoms, Pre-Appointment, Appointment, Details of Experience, and After Initial Appointment. 

### 2.2. Study Questionnaire

The online questionnaire was distributed from November 2022 to February 2023. Study participants were recruited through cancer advocacy groups, contacts at various Canadian cancer centres, and members of the EAP, who distributed the infographic and survey to eligible patients. CCC also shared a recruitment poster through various social media platforms (i.e., Instagram, Facebook, Twitter, LinkedIn) and their monthly newsletter. As an incentive, survey participants who fully completed the survey were offered entry into a random selection draw to win one of ten CAD 25 Amazon e-gift cards. Individuals were eligible to participate in the study if they met the following inclusion criteria: diagnosed with CRC within the past 10 years, aged 18 years or older, read and spoke English or French, resided in Canada, and provided virtual consent.

### 2.3. Statistical Methods

The extracted data were available in a Microsoft Excel format (CSV file) and cleaned for duplicate and/or non-qualified entries. The cleaning procedure utilized the Excel “sort” feature to screen for identical or nearly identical entries. The “sort” feature also filtered responses that did not meet the inclusion criteria, such as diagnoses that occurred more than 10 years prior. Of the 361 survey responses collected, Excel detected 39 that did not meet the inclusion criteria.

A total of 175 survey entries were analyzed. The N/A notation described blank answers detected during the analysis. French entries were carefully translated into English for analysis by a study team member proficient in both languages. A mixed-methods approach comprising quantitative and qualitative methods was used to analyze the data. The descriptive analysis portion of the study involved calculating measures of central tendency (e.g., mean, median, mode) and variability (e.g., standard deviation) for continuous variables and proportions (%) for categorical variables. Excel was used to calculate and present the results in tables, figures, and charts. Stratification was used to compare differences between groups (e.g., age groups, cancer types, and cancer stages). Moreover, SPSS 29 (quantitative data analysis software) was used to calculate Spearman rho associations between relevant ordinal variables and CRC awareness aspects. The significance was set a priori at *p* < 0.05. The qualitative portion of the study assessed the free-text responses featured in the survey. A total of 130 free-text responses from the open-ended question were analyzed through NVivo 12 (qualitative data analysis software). The Braun and Clarke framework was applied to the responses to inductively identify latent themes and patterns. Information regarding survey questions can be found in Supplementary Materials.

### 2.4. Ethics Approval

The study received ethics approval from the Human Research Ethics Board at the University of Waterloo (REB #44584).

## 3. Results

### 3.1. Quantitative Analysis

#### 3.1.1. Demographics

The demographic questions provided useful characteristics about the survey participants. This survey received 175 responses across Canada. The highest proportion resided in Ontario (38.9%), with the common community type being urban (49.7%) (Table A1). Meanwhile, 58.3% self-identified as women/female and 80.0% as White (Table A1). The age distribution varied amongst participants, with most respondents belonging to the 60+ year (26.3%) or 30–39 year (23.4%) age groups (Table A1). Most respondents (84.6%) reported having at least a college/CÉGEP diploma, and half of the respondents reported having a bachelor’s degree or higher (49.7%) (Table A1). 

#### 3.1.2. Cancer Sample

**Figure 2:**
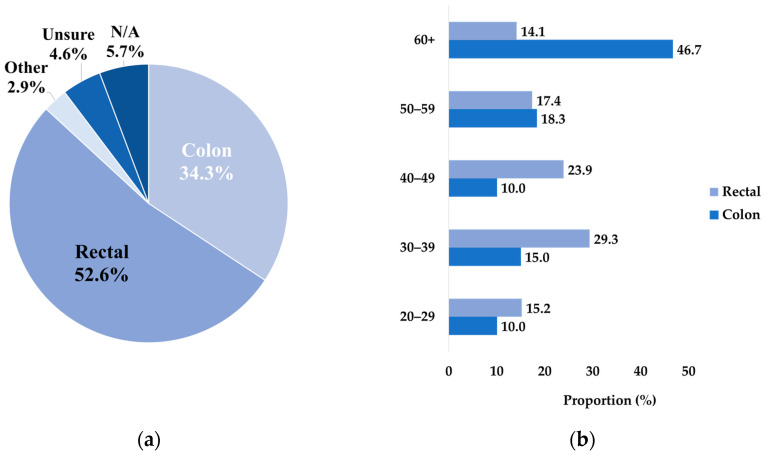

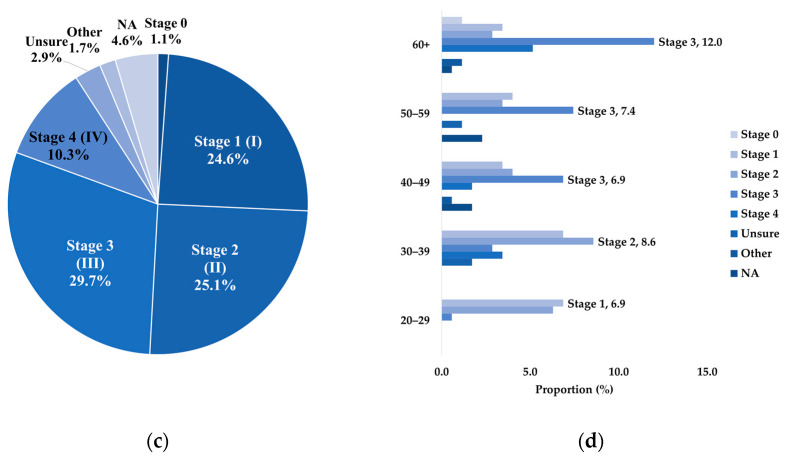
The cancer demographics of the participant pool. (**a**) The proportion of respondents who experienced colon cancer compared to rectal cancer. (**b**) The age stratification of the cancer type distribution. (**c**) The distribution of the cancer stage amongst the participant pool. (**d**) The age stratification of the cancer stage distribution.

Of the 175 participants, about half had rectal cancer (52.6%), and a third had colon cancer (34.3%) (Figure 2a). Stage 2 or 3 cancers were the prominent diagnosis stage for more than half of the participating patients (54.9%) (Figure 2c). A higher proportion of respondents who had colon cancer were Stage 3 (13.1% of total respondents), while a higher proportion of respondents with rectal cancer were Stage 1 (18.3%) (Table A2). 

Stratification of the cancer type by age and stage showed that the 50–59 year and 60+ year age groups had a higher proportion of colon cancer cases, while the 40–49 year and below age groups had a higher proportion of rectal cancer cases (Figure 2b). Interestingly, the highest proportion of participants for the 40 year and above age groups were diagnosed with Stage 3 cancer. In comparison, the highest proportions of participants from the 30–39 year and 20–29 year age groups were diagnosed with Stage 2 and Stage 1 cancer, respectively (Figure 2d).

#### 3.1.3. Access to Family Physicians: Barriers, Enablers, and Wait Times

**Figure A1:**
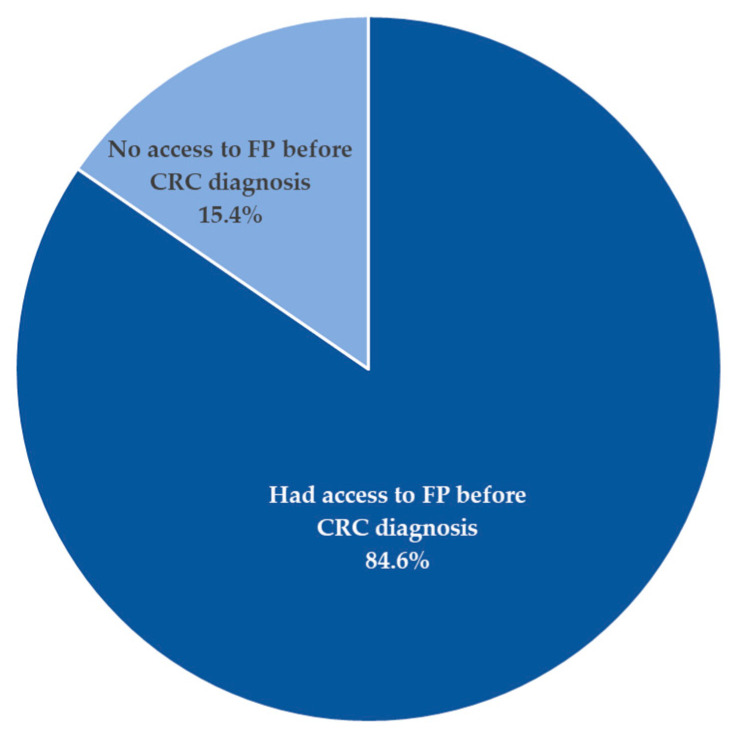
The proportion of respondents that had access to an FP before their diagnosis.

**Figure A2:**
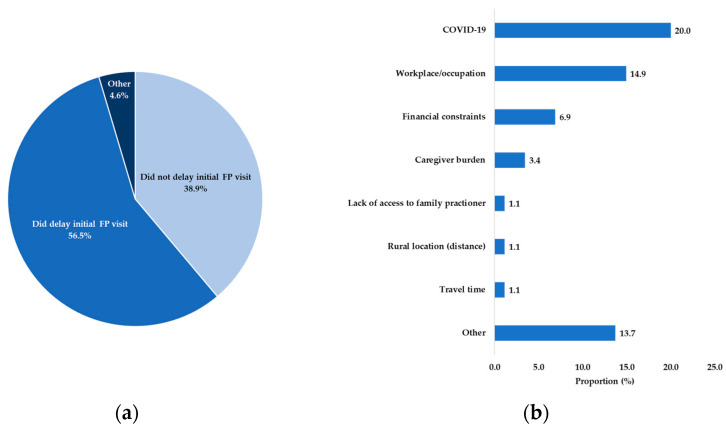
The distribution of barriers survey participants faced in accessing an FP. (**a**) The proportion of individuals that delayed their initial FP visit. (**b**) The proportion of the types of challenges that delayed respondents’ ability to obtain an FP appointment.

The primary endpoint of the study was FP accessibility. Most of the participants did have access to an FP before their CRC diagnosis (84.6%) (Figure A1). More than half of respondents did not delay their initial FP visit (56.6%). Of those respondents who did delay their visit (43.4% of total respondents), the highest proportion listed COVID-19 (20.0% of total respondents) or workplace/occupation (14.9%) as the reason (Figure A2).

Of the 175 respondents, 56.6% of respondents had their CRC diagnosis explained by an FP. Of these respondents, the vast majority felt fully informed by the FP’s explanation of their diagnosis (49.7% of total respondents) and had their diagnosis explained using plain language (53.1%). Respondents reported being given verbal explanations (31.1%) and/or visual explanations (31.1%); however, 40.6% of respondents did not receive any recommendations for CRC management. Only 17.7% were diagnosed with CRC by an FP; 38.3%had their diagnosis explained to them by another health care provider (Table A3).

The survey assessed wait times. An analysis based on average age showed that the average age at symptom onset was 41 ± 14 y/o (n = 113), at the initial FP appointment was 42 ± 15 y/o (n = 157), and at CRC diagnosis was 43 ± 15 y/o (n = 167) (Table 1). 

Other variables included wait times at different points throughout the diagnostic process. Before visiting an FP, about a third of the respondents (32.6%) experienced symptoms for 1–3 months, and 21.7% experienced symptoms for 3–6 months (Table 2). With respect to the time to obtain an appointment with an FP, most respondents acquired an appointment within less than a week (32.6%) or 1–2 weeks (36.6%) (Table 3). Patients typically had to wait less than three months to receive a referral (65.1% of total responses). The highest proportion of respondents waited 2–4 weeks for their screening test completion (31.4%), Around a third (31.3%) of patients only had to wait 1–2 weeks before receiving their screening results from their FP, while 16.0% had these results disclosed by a specialist. After initially seeking medical attention, the highest proportion of respondents waited less than a month until their FP suspected CRC (32.0%). A fifth of respondents (20.0%) had another health care provider suspect their CRC (Table A4). From the time the respondent initially sought medical assistance until the time of their CRC diagnosis, 38.3% of respondents waited 3 or more months and up to two years (Table 4). 

#### 3.1.4. Dismissal and Misdiagnoses of CRC

**Figure 3:**
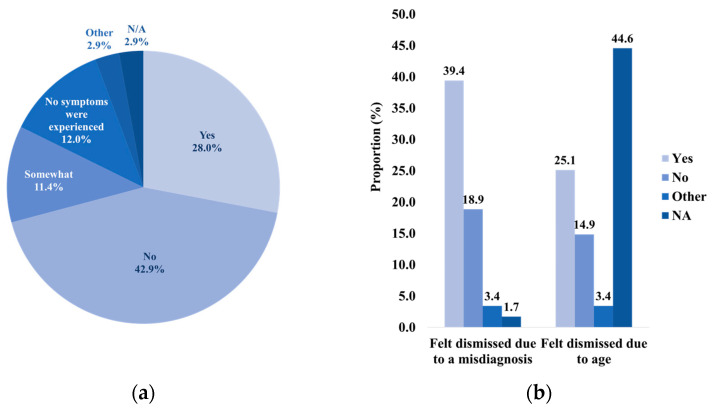
The distribution of sentiments of dismissal amongst survey participants. (**a**) The proportion of individuals who felt as if their symptoms were dismissed by their FP. (**b**) The proportion of those who felt dismissed or somewhat dismissed that experienced age-based dismissal and misdiagnosis-based dismissal. The highest proportion of N/A responses amongst those who felt age-based dismissal that could not be further explained by age stratification (Figure A4).

**Figure A3:**
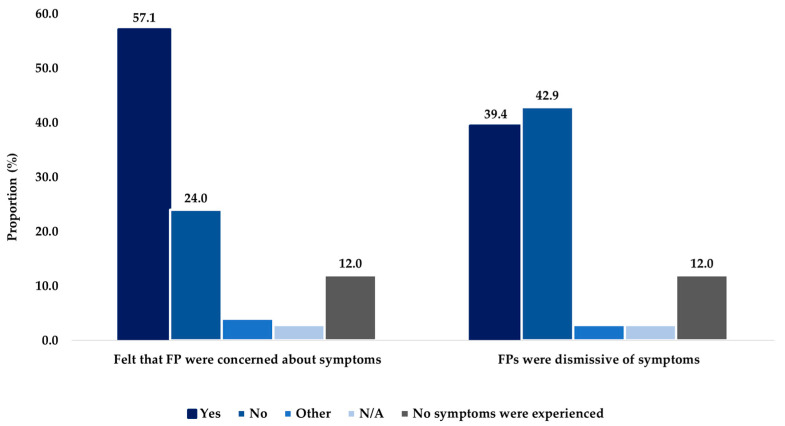
Concern from FP about symptoms and dismissal of symptoms.

**Figure A4:**
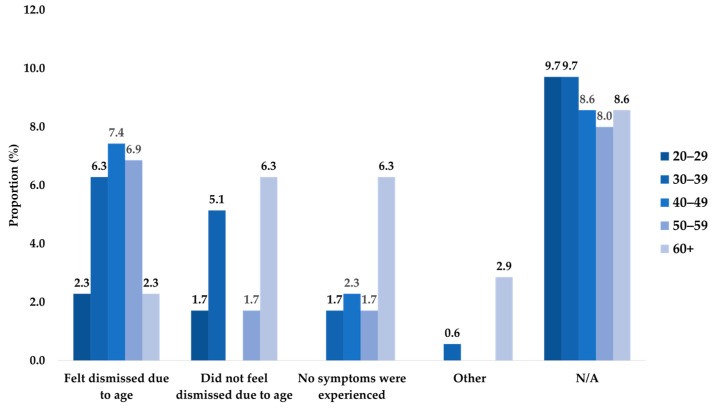
Age stratification of the dismissal due to age responses.

Another primary endpoint focused on the dismissal and misdiagnosis of CRC. It was found that 57.1% of respondents felt their FPs were concerned about their symptoms or results, and 42.9%, did not feel dismissed by their FP (Figure A3). Of the 39.4% of respondents who reported feeling dismissed or somewhat dismissed, many suspected they were dismissed due to their age (25.1% of total respondents) (Figure 3b). Stratification by age revealed that the highest proportion of those who felt age-based dismissal belonged to the 40–49 year age group (7.4% of total respondents) (Figure A4).

**Figure 4:**
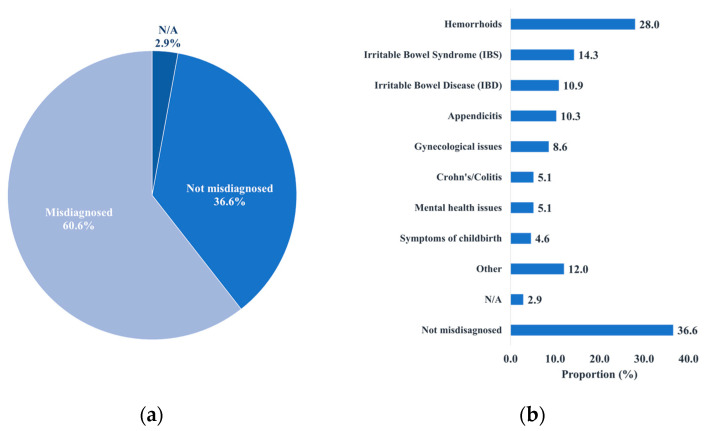

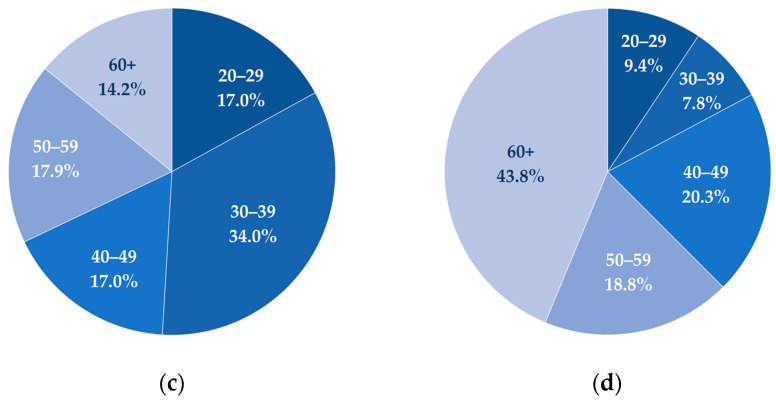
The distribution of misdiagnoses amongst survey participants. (**a**) The proportion of participants that were misdiagnosed. (**b**) The types of misdiagnoses that participants received. (**c**) The age stratification amongst participants that were misdiagnosed. (**d**) The age stratification amongst participants that were not misdiagnosed.

**Figure A5:**
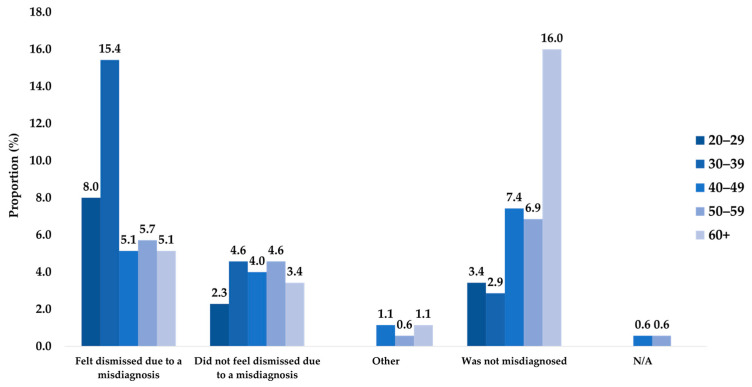
Age stratification of the dismissal due to misdiagnosis responses.

All respondents who reported feeling dismissed or somewhat dismissed suspected dismissal due to misdiagnosis (39.4% of total respondents) (Figure 3b). Age stratification highlighted that the highest proportion that experienced misdiagnosis-based dismissal belonged to the 30–39 year age group (15.4% of total respondents) (Figure A5). Based on the survey responses, 60.6% reported misdiagnoses throughout their experience (Figure 4a). The highest proportion of those not misdiagnosed were part of the 60+ year age group (16.0% of total respondents), and the highest proportion of those misdiagnosed were part of the 30–39 year age group (20.6% of total respondents) (Figure 4c,d). The most common misdiagnosis received was that of hemorrhoids (28.0%). Only 36.6% of respondents were not misdiagnosed (Figure 4b). 

#### 3.1.5. CRC Awareness

**Figure A6:**
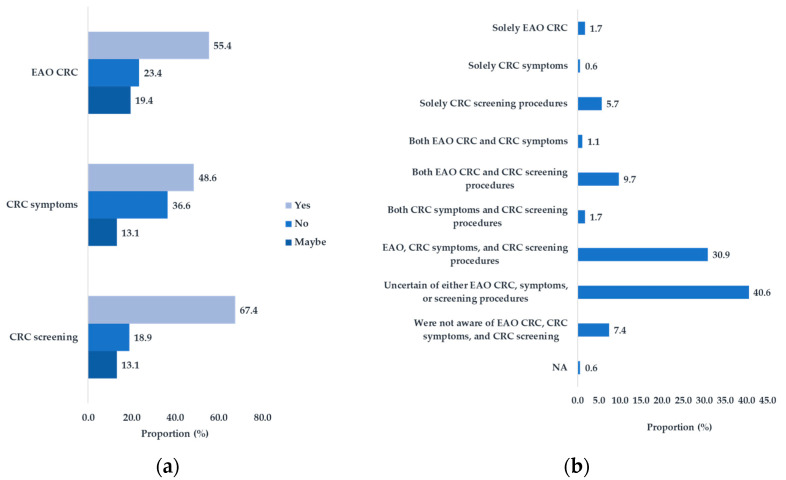
The distribution of pre-diagnosis CRC awareness. (**a**) The proportions of respondents that were aware of different CRC aspects. (**b**) The combined distribution of respondent awareness of CRC aspects.

The secondary endpoint of the study was CRC awareness prior to diagnosis, including awareness of symptoms and screening and awareness that CRC could occur in those aged 50 years and younger (i.e., EAO CRC). Interestingly, 30.9% of respondents had knowledge of all three prior to diagnosis, while 40.6% of respondents were uncertain of at least one of these aspects of CRC awareness (Figure A6a). Specifically, 48.6% knew of CRC symptoms, 67.4% of the 175 participants were previously aware of CRC screening methods (67.4%), and 55.4% knew of EAO CRC (Figure A6b). 

A Spearman’s rho association analysis revealed a statistically significant, positive but weak correlation of 0.243 [95% CI 0.086,0.388, *p* = 0.008] between patients with no prior knowledge of symptoms and the time until diagnosis after initially seeking medical attention. Furthermore, the analysis also revealed a statistically significant, negative but weak correlation of 0.172 [95% CI −0.323, −0.012, *p* = 0.030] between patients with CRC symptom awareness and the time until diagnosis after initially seeking medical attention.

#### 3.1.6. Pre-Diagnosis Experience

**Figure 5:**
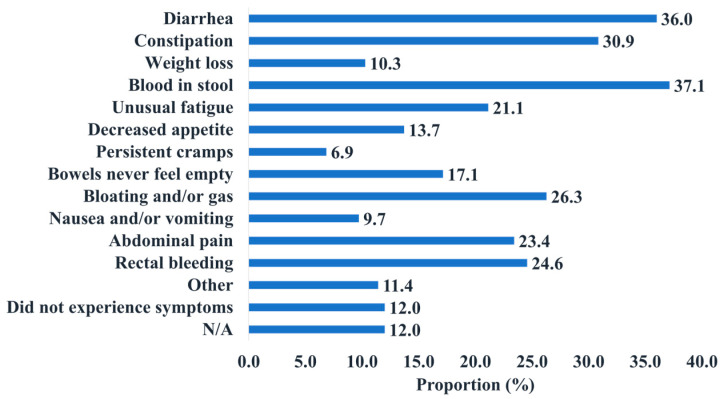
The distribution of the types of CRC symptoms the participants experienced.

**Figure A7:**
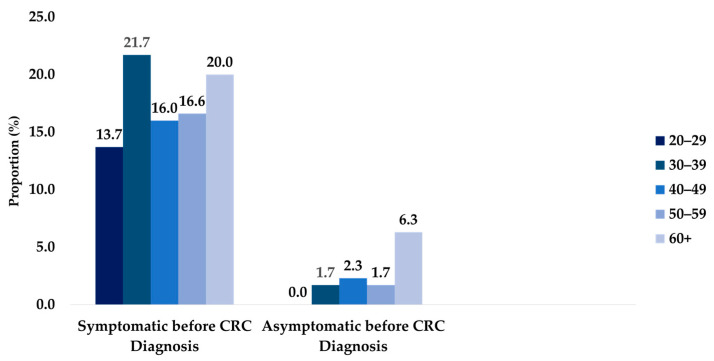
The age distribution of the participants that did and did not experience CRC symptoms before their diagnosis.

The pre-diagnosis portion of the survey assessed the period between first experiencing symptoms up until the time of specialist referral. Of the 175 participants, 88.0% experienced symptoms. The most common symptom reported by participants was blood in the stool (37.1%) and diarrhea (36.0%) (Figure 5). Amongst the 88% that experienced symptoms, more individuals suspected that these symptoms were not CRC-related (38.3% of the total respondents) as compared to those who suspected that these symptoms were CRC-related (34.3% of the total respondents). Around half inquired whether the symptoms were in relation to CRC (48.6% of total respondents). Only 18.9% of respondents sought their initial FP visit through a routine screening procedure, while the majority (73.1%) did not (Table A5). Meanwhile, 12% of the participants described themselves as being asymptomatic. The highest proportion of those who were asymptomatic belonged to the 60+ year age group (6.3% of total participants) (Figure A7), while the higher proportion of symptomatic participants belonged to the 40–49 year and below age groups (51.4% of total participants) (Figure A7).

**Figure A8:**
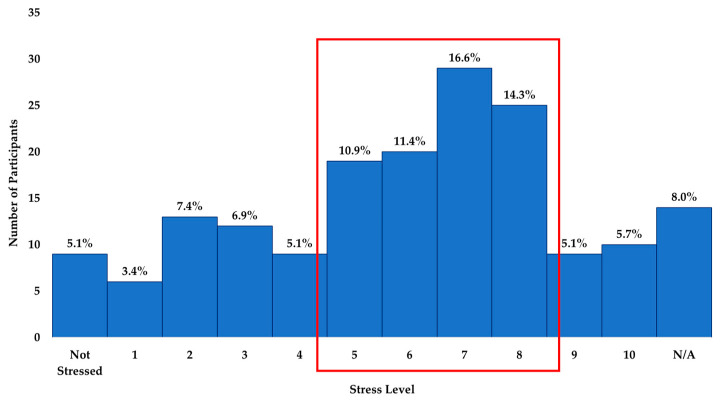
Distribution of stress amongst respondents leading up to initial CRC FP appointment. The red box indicates that 53.2% of respondents experienced stress levels within the 5–8 range.

During the wait for the initial FP appointment, 31.4% of respondents experienced a worsening of symptoms, amongst whom a small proportion sought emergency care (5.7% of total respondents) (Table A6). The average stress level amongst participants was 6 (±3 OR SD = 3) out of 10, with 10 meaning a very high level of stress and 0 meaning no stress. Half of the respondents experienced stress levels within the moderately high, 5–8 range (53.1%) (Figure A8). 

Of the 175 participants, 40.6% of respondents did not discuss CRC risk factors with family members or their FPs. Only 58.3% reported being asked about their family history of CRC or polyps by the FP. Further, only 32.0% underwent genetic testing through their FP. Meanwhile, 66.9% were referred to a single specialist, with the highest proportion being referred to a gastroenterologist (56.0% of the total respondents) (Table A7).

#### 3.1.7. Eventual and Post-Diagnosis Experience

The eventual and post-diagnosis portion of the study describes patient sentiments throughout screening, diagnostic testing, and subsequent care. Regarding screening, 47.4% underwent multiple screening tests. The most common screening tests recommended by FPs reported amongst participants were colonoscopies (54.9%), followed by the FIT (45.7%). A high proportion of respondents discussed their screening results with an FP (62.9%). In terms of diagnostic testing, 61.1% of participants underwent a colonoscopy. Additionally, 31.4% of respondents reported that they paid for private testing in order to receive their CRC diagnosis (Table A8), while the highest proportion of respondents had two appointments until their FP suspected CRC (23.4%). 

**Figure A9:**
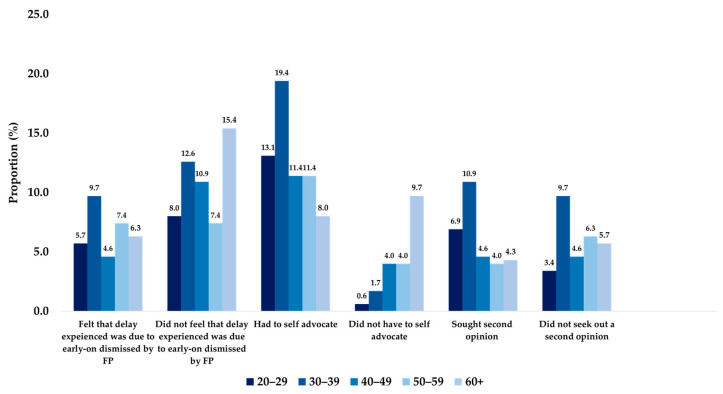
The age distribution of different post-CRC diagnosis patient experiences.

Out of 175 respondents, 72.6% felt that their CRC was not taken seriously and not diagnosed in time. Around a third felt that their CRC diagnosis was delayed due to dismissal by their FP early on (33.7%). When respondents were asked about potential reasons as to why they felt as if their CRC was not taken seriously, most reported “not having a family history of CRC” (34.9%) as a reason. Furthermore, a high proportion of respondents who experienced symptoms had to self-advocate for their diagnosis to be taken seriously (63.4% of total respondents) (Table A9). The 30–39 year age group made up the highest proportion of those who had to self-advocate (19.4% of total respondents). In comparison, the 60+ year age group made up the highest proportion of the 20.0% that did not self-advocate. Of the participants who experienced misdiagnoses (60.6%), around half sought a second opinion (29.7% of total respondents) (Figure A9). 

### 3.2. Thematic Analysis

**Figure 6:**
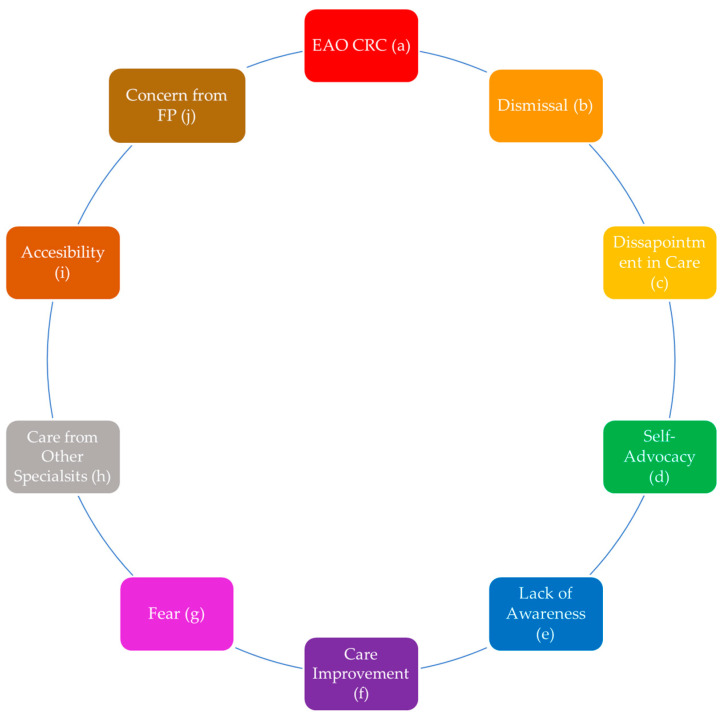
Key themes associated with Canadian patient experiences with FPs throughout their CRC diagnosis journey. The boxes highlight quotes exemplifying the themes of (**a**) EAO CRC, (**b**) dismissal, (**c**) disappointment in care, (**d**) self-advocacy, (**e**) lack of awareness, (**f**) care improvement, (**g**) fear, (**h**) care from other specialists, (**i**) accessibility, and (**j**) concern from the FP.

The open-text responses provided in the survey were categorized into ten themes (Figure 6). Qualitative data that were collected highlighted multiple gaps in CRC care.

#### 3.2.1. EAO CRC and Dismissal

The EAO CRC theme developed from patient responses where younger individuals felt dismissed by their FP due to their age. These younger patients expressed that only upon their insistence or symptom worsening would their concerns be investigated. For example, one young patient mentioned the following:

“I feel there was a delay in my care because it took a while for me to assert myself with my GP. No blame on him but I do feel once I told his resident I saw blood then things happened fast. I regret that as a “young” person I had no clue what colon cancer was. Didn’t think it happened to young people. Was not on my radar at all.”

(Figure 6a).

Symptom dismissal was found to be a common theme regardless of age. In these cases, patients were either deemed healthy, were dismissed due to mild symptoms, or were misdiagnosed altogether. Patients reported that because of these presumptions, symptoms worsened, and their diagnosis was delayed. For example, one patient expressed the following:

“I had extreme cramps so went to emergency where I was told it was menstrual. I then had severe chest pain on two occasions that required going to the ER. It was only on the second visit that they did a CT scan and found liver lesions.”

(Figure 6b).

This consistent scenario showed that patients were not being taken seriously and in turn, suffered.

#### 3.2.2. Lack of Awareness and Fear

Many patients admitted to having a lack of prior knowledge of different aspects of CRC. This lack of awareness prevented them from receiving timely treatment during the diagnostic process. They expressed that they were unable to self-identify their symptoms and therefore had to self-advocate. One patient described being “grateful I finally went. Have no family history and had yearly FoBt tests. Thought my issue was a prolapse or rectocele or IBS. He sent me for urgent scope and was so right to do so.” (Figure 6e). Patients also divulged that feelings such as fear and embarrassment caused them to refrain from seeking out care, and many of these respondents only sought care once symptoms worsened. One patient shared the following: “I initially kept it to myself. Symptoms gradually got worse and blood in stool became more frequent before going to a doctor.” (Figure 6g).

#### 3.2.3. Disappointment in Care and Self-Advocacy

A proportion of patients described dismay with respect to the primary care they received. These patients reported feeling isolation and a lack of support, that their concerns were not appropriately addressed, and that the continuity of care did not continue throughout the referral process. One patient indicated the following:

“I don’t feel that my family doctor ever took it seriously and that it took far too long to come to the proper diagnosis. I was lucky in that it did not spread but for someone else 2 years could have meant their life. He did not contact me after diagnosis either. I feel that my care was not handled well by him. From him I received no follow up instructions or supports.”

(Figure 6c).

A theme of self-advocacy became apparent from responses detailing that respondents had to urge their care provider to take their symptoms seriously. Respondents reported doing their own research prior to seeing their FP and bypassing their FP altogether by seeking out emergency or private care. One patient expressed that there was a “lack of information given throughout the whole adventure.” This patient continued as follows: “I gather I was expected to do all the research and answer my own questions through google.” (Figure 6d). Responses reflected that patients felt they could not depend on their FPs.

#### 3.2.4. Care Improvement and Accessibility

Patients made recommendations for changes they would like to see implemented for the future management of CRC. The suggested changes including better technology and facilities, more efficiency (i.e., quicker turnover for all steps in the diagnosis journey), and an overall need for more support and awareness about CRC. Some respondents also commented on increased accessibility to private testing and less dependency on virtual care. One patient mentioned, “I’m 80 years old. I had a family doctor for at least 20 years, never discussed preventive colonoscopy.” (Figure 6f). Responses also detailed various delays that were experienced throughout the diagnostic process. As expected, COVID-19 was viewed as a contributing factor. In addition, patients felt that FPs were too busy and that having to go through multiple tests and/or referrals contributed to slow turnovers and a lack of oversight throughout the diagnostic process. One patient stated,

“My doctor was good, gave me the FIT test kit and booked colonoscopy, COVID-19 delayed the colonoscopy by 3 months, but after a positive colonoscopy It took less than 3 weeks to meet the surgeon and surgery was booked within the next month.”

(Figure 6i).

#### 3.2.5. Concern from FPs and Care from Other Specialists

Patients described positive experiences with their FPs, which included informative explanations, slow and thorough answers to all posed questions, and timely and appropriate investigations. One patient appreciated that their FP took,

“The time to explain the necessity of the routine FIT test and making sure I completed it and promptly referring me for further treatment, he saved my life. The cancer might have been a lot less treatable if I had waited until symptoms developed”.

(Figure 6j).

Many of these responses highlighted the significant role of FPs in providing access to routine testing/procedures. Respondents also reported mainly positive experiences with medical specialists. These reflections provided insight into the limitations of FP care and the need for a transition of care from FPs to more specialized providers. For example, a patient stated that their FP did the following:

“Encouraged an early colonoscopy due to family history, but otherwise was not involved in the diagnosing and treatment of my CRC, as the urgent care I received from the gastro, surgeon, and oncologist covered all bases.”

(Figure 6h).

## 4. Discussion

This study highlights gaps within the CRC care continuum in Canada based on patient experiences. The findings suggest delays in diagnosis due to limited awareness of CRC screening and symptoms, as well as barriers in accessing timely diagnostic tests (e.g., colonoscopies). Eventual diagnosis experiences emphasize the need for a patient-centred approach for patients to feel fully informed about their CRC diagnosis. Shared patient experiences highlight difficulties in identifying CRC symptoms amongst younger people, resulting in dismissals and misdiagnoses.

The survey results suggest that the pre-diagnosis awareness of CRC affects the diagnostic process. Studies have found that being unaware of CRC symptoms can lead to delays in diagnosis, and the Spearman ⍴ analysis conducted supports this finding [12,13]. Patient testimonies expanded on this point, describing that having no prior knowledge of CRC made it difficult for them to advocate for themselves or self-identify symptoms, delaying their diagnosis. To address awareness gaps, general educational campaigns (e.g., mass media campaigns) have been shown to be effective in increasing awareness of CRC symptoms and screening [14].

Although most respondents had access to FPs before their CRC diagnosis, patients reported challenges before being initially seen. Interestingly, more than half of the participants fell within the lowest wait-time categories of the periods analyzed. However, this proportion only represented a fraction of participants and still described a 2 to 7-month wait from symptom onset to diagnosis. A study found that delayed diagnosis and treatment are not associated with increased mortality amongst symptomatic CRC patients [15] although early detection and treatment could play a key role in reducing morbidity and mortality amongst asymptomatic CRC patients [15]. However, the staging of the disease might be impacted by a later diagnosis, as longer diagnostic intervals are associated with more advanced CRC [16]. Similarly, one respondent indicated that if their FP had listened to their concerns and screened them earlier, they would not have received a later-stage CRC diagnosis. Regardless, these delays should be prevented to reduce the psychological impact amongst all patients [15,17].

Along with delays, the survey also revealed differences in the support provided throughout the CRC diagnostic process. Feeling unsupported and isolated were common sentiments emphasized by patients. Although respondents had access to FPs, not all were initially thought to have CRC, and when their diagnosis was explained to them, they did not feel fully informed. By considering different levels of patients’ health literacy, FPs can build trust amongst patients over time by using diverse communication strategies, adjusting language, repeating explanations, and involving caregivers [18].

Additionally, patients felt as if they had to do their own research since their FPs were unable to provide the answers that they needed. Respondents reported a lack of recommendations from FPs for managing their CRC, and only a small proportion of respondents reported being offered resources (e.g., educational materials) about CRC. Multiple studies have shown that collaborative communication styles that apply empathy, respect, and less verbal dominance positively influence diagnostic efficiency and patient health outcomes [19,20,21]. Additionally, improving patient-centred care (PCC) would also improve diagnostic pathways for CRC patients. Similarly, one respondent indicated that their FP saved their life by taking the time to explain the importance of a routine FIT test and ensuring the patient received prompt referral for further treatment. This is important because timely and efficient diagnosis leads to better patient outcomes [22].

A review article on the pitfalls and missed opportunities in the process of CRC diagnosis in symptomatic patients found that the vagueness and non-specific nature of CRC symptoms, combined with poor awareness of the disease amongst patients and FPs, often led to patients dismissing their symptoms or being misdiagnosed by a health care provider [23]. Also, a lack of family history was the most commonly reported reason for diagnoses not being taken seriously. As a result of these gaps, delays in the diagnostic process are common [24]. To address this challenge, it is necessary to promote the collection of family history (CRC and polyps) in primary care, especially amongst younger adults, to better inform screening and referral for risk assessment [25].

Moreover, many participants reported feeling moderately to highly stressed about their initial FP appointment. In-person appointments were reported to be more common than were virtual appointments. While in-person care has been viewed as the “gold standard” for patient–physician interaction, virtual care, such as telemedicine, can help overcome the challenge of geographical distance for patients in remote or rural regions and reduce anxiety amongst cancer patients [26,27]. In one study, it was found that replacing traditional in-person care with technology-based remote options can lead to improvement in the psychosocial health outcomes (e.g., anxiety, depression,) associated with cancer diagnosis and treatment [27]. 

Additionally, private and genetic testing for CRC diagnosis was uncommon, and few participants reported that their FP or other HCPs ordered genetic testing. One study found that there are disparities in accessing genetic/genomic testing in various provinces across Canada due to the lack of published standards for HCPs, adequate infrastructure, and resources/funding [28]. This underscores the need for standardized genetic/genomic testing approaches across Canada to ensure patients have equitable and timely access to testing [28].

Furthermore, it was reported that FPs most commonly utilized colonoscopies and FIT tests for CRC screening, while colonoscopies were the most preferred diagnostic test. A growing demand for these tests is a challenge due to limited resources and waiting periods [23]. Some participants reported waiting a long period to obtain a colonoscopy due to various accessibility challenges, such as COVID-19 delays and dismissals from FPs. In Canada, the average wait time for colonoscopies due to a positive FIT was reported to be 76 days in 2022 [29]. Timely access to colonoscopies is crucial, as prolonged wait times can delay diagnosis and treatment, impacting patient outcomes.

Amongst participants who experienced symptom dismissal, age-based dismissal was common. To address these challenges, it is important to sensitize FPs (e.g., offer educational workshops) to aspects of CRC, which can promote earlier detection and reduce misdiagnoses and EAO CRC cases. Earlier detection in younger populations can also be promoted by lowering the floor of Canadian CRC screening age to 45 years [30].

Further, based on milestone markers highlighted in the survey, on average, participants experienced their CRC diagnostic process during their early 40s and the process took 1–2 years from symptom onset. Similarly, studies have found that younger age groups face more difficult diagnostic paths and less positive diagnosis-related care experiences than do older age groups [31,32,33]. The observed trends concerning EAO cancers suggest a need to encourage symptom awareness, screening, and seeking of medical aid amongst younger individuals to improve upon earlier detection [25]. These trends also help to inform FPs of age-related barriers throughout the cancer referral pathway [25].

Due to limitations of the recruitment methods and study design, patient behavior could have potentially biased the results obtained (e.g., recall bias and non-response bias) and impacted the internal validity of the study. This study included participants who were diagnosed up to 10 years prior, so participants might have inaccurately remembered past events or experiences, potentially leading to recall bias.

Moreover, the study was reviewed by the CCC team and an EAP of family physicians, but a validation study for the questionnaire was not conducted amongst the target population, which may limit the reliability (e.g., internal consistency) and validity of the questionnaire, such as the construct and criterion validity—thus limiting the research findings.

Although inferential statistics were conducted during the analysis, a sensitivity analysis could not be done to determine the influence of bias on the results, and most of the correlations generated were found to be non-significant (*p* > 0.05). Moreover, the sample size was relatively small (175 responses) and reflected self-reported information. Thus, there is potential for confounding factors and effect modifiers such as age, gender, and level of education, which may impact the validity and reliability of the correlations presented (Supplemental Material Table S1). Additionally, the small sample size limits the generalizability of the findings to the broader population.

The respondent pool may not entirely reflect a cross-section of the overall population of Canada who have had colorectal cancer, which might have led to non-response bias. Based upon the self-reported demographic information, respondents tended to have higher levels of education, live in urban communities, identify as being White, be aged 30+ years, and identify as being female. Additionally, due to the eligibility criteria, the study might have excluded patients who did not have access to a device (to complete the survey) or were unable to communicate in either English or French. The perspective of the responses was also skewed towards a rectal cancer patient’s experience. These criteria must be taken into consideration when applying the study results to the broader population. 

## 5. Conclusions

This study uncovered significant gaps in the CRC care pathway in Canada, as highlighted by patient experiences throughout their diagnostic process. It emphasized the critical role of pre-diagnosis awareness and the impact of limited knowledge about CRC symptoms on diagnosis delays. Moreover, it pinpointed accessibility barriers in obtaining a timely diagnosis and insufficient support from FPs, resulting in patients not feeling fully informed about their CRC diagnosis. Additionally, this study highlighted delays and disparities in accessing essential diagnostic tests such as genetic testing and colonoscopies. Particularly, shared patient experiences brought attention to challenges in recognizing CRC symptoms amongst younger individuals, leading to dismissals and misdiagnoses. Future research should be conducted to explore the challenges that FPs encounter with respect to the prompt diagnosis and referral of patients with CRC. Additionally, more in-depth research should explore age-related discrepancies in CRC care; barriers and enablers in accessing FPs; and reasonings behind symptom dismissal and misdiagnosis amongst different populations (e.g., rural populations and different ethnic groups). These insights will be instrumental in developing educational resources and targeted recommendations for both patients and FPs, aimed at enhancing early CRC detection, streamlining referral processes, and ultimately improving patient outcomes. This study may also help inform policies aimed at improving access to timely and accessible care—and lead to better care initiatives for all CRC patients.

## Figures and Tables

**Table 1:** Patients age at different points throughout their CRC diagnosis journey.

Measure	Age at Symptom Onset (y)	Age at Initial FP CRC Visit (y)	Age at CRC Diagnosis (y)
Mean m ± s.d.	41 ± 14	42 ± 15	43 ± 15
Median N (Q1, Q3)	40 (30, 52)	41 (29, 53)	43 (30, 53)
Mode	45	28	30
Range [Min, Max]	(12, 79)	(18, 79)	(18, 80)

**Table 2:** The length of time patients experienced symptoms prior to their CRC diagnosis.

Duration	Number of Respondents	Proportion (%)
Less than 1 month	23	13.1
1–3 months	57	32.6
3–6 months	38	21.7
6–12 months	17	9.7
Greater than 1 year	16	9.1
No symptoms were experienced	21	12.0
Other	3	1.7

**Table 3:** The length of time patients waited to obtain an initial FP appointment prior to CRC diagnosis.

Duration	Number of Respondents	Proportion (%)
Less than 1 week	57	32.6
1–2 weeks	64	36.6
3 weeks	23	13.1
4–6 weeks	11	6.3
Greater than 6 weeks	4	2.3
Other	13	7.4
N/A	3	1.7

**Table 4:** The length of time patients waited from symptom onset to their CRC diagnosis.

Duration	Number of Respondents	Proportion (%)
Less than 1 month	51	29.1
1–3 months	49	28.0
3–6 months	30	17.1
6–12 months	17	9.7
Greater than 1 year	12	6.9
Other	8	4.6
N/A	8	4.6

## Data Availability

The data used in this study are not publicly available; however, they may be available upon request. Please contact the corresponding author.

## Supplementary Materials

The following supporting information can be downloaded at https://www.mdpi.com/article/10.3390/curroncol31060237/s1, Table S1: All Spearman’s rho (ρ) associations tested using SPSS.

## Appendix A

**Table A1:** Demographics of the survey pool.

Demographic Variable	Number of Participants (n)	Proportion (%)
Province or Territory		
Alberta	22	12.6
British Columbia	21	12.0
Manitoba	3	1.7
New Brunswick	6	3.4
Newfoundland and Labrador	11	6.3
Northwest Territories	1	0.6
Nova Scotia	11	6.3
Nunavut	4	2.3
Ontario	68	38.9
Prince Edward Island	2	1.1
Quebec	22	12.6
Saskatchewan	3	1.7
Yukon	1	0.6
Ethnicity/Race		
Arab	4	2.3
Black	6	3.4
Chinese	1	0.6
Filipino	2	1.1
Japanese	2	1.1
Latin American	5	2.9
First Nations, Métis, Inuit	5	2.9
South Asian (e.g., East Indian, Pakistani, Sri Lankan)	1	0.6
White	140	80.0
≥2 Races/Ethnicity	8	4.6
Prefer not to answer	1	0.6
Gender/Sex		
Man/Male	70	40.0
Women/Female	102	58.3
Trans/Non-binary	2	1.1
N/A	1	0.6
Current Age		
20–29 years	24	13.7
30–39 years	41	23.4
40–49 years	32	18.3
50–59 years	32	18.3
60+ years	46	26.3
Community Type		
Urban	87	49.7
Suburban	56	32.0
Rural	32	18.3
Educational Level		
Less than high school diploma	1	0.6
High school diploma or equivalent	26	14.9
CÉGEP	5	2.9
College certificate/diploma/trade college	56	32.0
Bachelor’s degree	57	32.6
Postgraduate certificate/diploma	7	4.0
Juris doctor (JD) degree	2	1.1
Master’s degree	18	10.3
Doctorate	3	1.7
Language Preference		
English	162	92.6
French	13	7.4

**Table A2:** Type and Stage of cancer amongst the survey pool.

Type of Cancer	Cancer Stage	Number of Participants (n)	Proportion (%)
Colon	Stage 0	2	1.1
	Stage 1 (I)	8	4.6
	Stage 2 (II)	11	6.3
	Stage 3 (III)	23	13.1
	Stage 4 (IV)	13	7.4
	Unsure	2	1.1
	Other	1	0.6
Rectal	Stage 0	0	0.0
	Stage 1 (I)	32	18.3
	Stage 2 (II)	27	15.4
	Stage 3 (III)	29	16.6
	Stage 4 (IV)	3	1.7
	Unsure	1	0.6
	Other	0	0.0
Other	Stage 1 (I)	1	0.6
	Stage 2 (II)	1	0.6
	Stage 4 (IV)	1	0.6
	Other	2	1.1
Unsure	Stage 1 (I)	2	1.1
	Stage 2 (II)	3	1.7
	Stage 4 (IV)	1	0.6
	Unsure	2	1.1
N/A	Stage 2 (II)	2	1.1
	N/A	8	4.6

**Table A3:** Enablers in FP accessibility through the CRC diagnosis process.

Attribute	Number of Participants (n)	Proportion (%)
Type of Professional that Diagnosed Respondent’s CRC		
Family practitioner	31	17.7
Emergency room (ER) doctor	29	16.6
Gastroenterologist	88	50.3
Oncologist	26	14.9
Obstetrician-gynecologist (OBGYN)	5	2.9
Urologist	6	3.4
Pediatrician	0	0.0
Surgeon	52	29.7
Other	3	1.7
N/A	8	4.6
Type of Explanations Offered by FP		
Verbal explanation	58	33.1
Visuals (e.g., diagrams/photos)	58	33.1
Resources (pamphlets, booklets)	22	12.6
Referral to Colorectal Cancer Canada Website	10	5.7
Other	1	0.6
A different health care provider explained diagnosis	67	38.3
N/A	9	5.1
FP Used Plain Language to Discuss CRC Diagnosis		
Yes	93	53.1
No	6	3.4
Other	1	0.6
A different health care provider explained diagnosis	67	38.3
N/A	8	4.6
Respondents Understood and Felt Fully Informed About Their CRC Diagnosis When It Was Explained by Their FP		
Yes	87	49.7
No	12	6.9
Other	1	0.6
A different health care provider explained diagnosis	67	38.3
N/A	8	4.6
Type of Recommendations Offered by FP		
Pharmaceutical (e.g., medications)	68	38.9
Resources (e.g., educational materials)	34	19.4
Referral to social worker	9	5.1
Support groups	17	9.7
Other	4	2.3
FP did not recommend anything	71	40.6
N/A	10	5.7

**Table A4:** Wait times experienced during the CRC diagnosis process.

Time Periods	Number of Participants (n)	Proportion (%)
Duration symptoms experienced prior to CRC diagnosis ^1^		
Less than 1 month	23	13.1
1–3 months	57	32.6
3–6 months	38	21.7
6–12 months	17	9.7
Greater than 1 year	16	9.1
Did not experience symptoms	21	12
Other	3	1.7
Time waited to obtain an initial FP appointment prior to CRC diagnosis ^1^		
Less than 1 week	57	32.6
1–2 weeks	64	36.6
3 weeks	23	13.1
4–6 weeks	11	6.3
Greater than 6 weeks	4	2.3
Other	13	7.4
N/A	3	1.7
Time waited to obtain a referral		
Less than 1 month	77	44.0
1–3 months	37	21.1
3–6 months	17	9.7
6–12 months	8	4.6
Greater than 1 year	8	4.6
Other	8	4.6
Not referred by a family practitioner	18	10.3
N/A	2	1.1
Time waited until screening results retrieved		
Less than 1 week	45	25.7
1–2 weeks	55	31.4
3 weeks	22	12.6
4–6 weeks	10	5.7
Greater than 6 weeks	1	0.6
Other	6	3.4
Other health care provider provided test results	28	16.0
N/A	8	4.6
Time waited from initially seeking help until FP suspected CRC		
Less than 1 month	56	32.0
1–3 months	39	22.3
3–6 months	16	9.1
6–12 months	2	1.1
Greater than 1 year	4	2.3
Other	14	8.0
Other health care provider suspected CRC	35	20.0
N/A	9	5.1
Time waited from symptom onset to CRC diagnosis ^1^		
Less than 1 month	51	29.1
1–3 months	49	28
3–6 months	30	17.1
6–12 months	17	9.7
Greater than 1 year	12	6.9
Other	8	4.6
N/A	8	4.6

^1^ These milestones are presented as separate tables in the main text.

**Table A5:** Pre-diagnosis CRC experience.

Attribute	Number of Participants (n)	Proportion (%)
Suspected symptoms were related to CRC		
Yes	60	34.3
No	67	38.3
Other	4	2.3
Did not experience symptoms	21	12.0
N/A	23	13.1
Inquired whether symptoms were related to CRC		
Yes	85	48.6
No	58	33.1
Did not experience symptoms	21	12.0
Other	7	4.0
N/A	4	2.3
Sought an FP appointment after a routine screening procedure		
Yes	33	18.9
No	128	73.1
Other	12	6.9
N/A	2	1.1

**Table A6:** Pre-diagnosis CRC symptom worsening and ER care-seeking experience.

While Waiting for an Appointment		Number of Participants (n)	Proportion (%)
Experienced symptom worsening	Sought ER Care		
Yes	Yes	10	5.7
	No	45	25.7
No	Yes	4	2.3
	No	80	45.7
No symptoms experienced	Yes	0	0.0
	No	18	10.3
	N/A	3	1.7
Did not wait for an appointment	N/A	14	8.0
N/A	N/A	1	0.6

**Table A7:** FP appointment experience before CRC diagnosis.

Variable	Number of Participants (n)	Proportion (%)
Discussed family health history, including CRC risk factors, before CRC diagnosis		
Only with family member(s)	22	12.6
Only with FPs	31	17.7
With both family member(s) and FPs	51	29.1
Did not discuss CRC risk factors	71	40.6
FP visit accompaniment		
Partner/spouse	59	33.7
Friend	19	10.9
Sibling	2	1.1
Child(ren)	0	0.0
Went alone	88	50.3
Other	3	1.7
N/A	4	2.3
Virtual appointment		
Yes	68	38.9
No	103	58.9
Other	1	0.6
N/A	3	1.7
FP asked about family history of CRC or polyps		
Yes	102	58.3
No	60	34.3
Other	8	4.6
N/A	5	2.9
FP ordered genetic testing for CRC		
Yes	56	32.0
No	92	52.6
Did not require genetic testing	7	4.0
Other health care provider ordered genetic testing	15	8.6
N/A	5	2.9
Number of specialists that FP referred respondents to		
1	117	66.9
2	32	18.3
3	6	3.4
Not referred by FP	18	10.3
N/A	2	1.1
Specialists referred by FP		
Emergency room (ER) doctor	16	9.1
Gastroenterologist	98	56.0
Oncologist	28	16.0
Obstetrician-gynecologist (OBGYN)	6	3.4
Urologist	5	2.9
Pediatrician	1	0.6
Surgeon	38	21.7
Other	7	4.0
Not referred by FP	18	10.3
N/A	2	1.1

**Table A8:** Types of screening and diagnostic tests patients experienced throughout CRC care pathway.

Variable	Number of Participants (n)	Proportion (%)
Number of screening tests		
1	73	41.7
2	44	25.1
3	23	13.1
4	11	6.3
5	3	1.7
6	0	0.0
7	2	1.1
Another health care provider ordered screening test(s)	16	9.1
N/A	3	1.7
Screening test offered by FP for CRC		
Fecal immunochemical test (FIT)/fecal occult blood test (FOBT)/immunochemical fecal occult test (IFOBT)	80	45.7
Colonoscopy	96	54.9
Digital rectum exam	45	25.7
CT colonography	28	16.0
Flexible sigmoidoscopy	8	4.6
Blood tests	36	20.6
Biopsy	6	3.4
Other	4	2.3
Another health care provider ordered screening test(s)	16	9.1
N/A	3	1.7
FP discussed results		
Yes	110	62.9
No	28	16.0
Unsure	3	1.7
Another health care provider discussed test results	27	15.4
N/A	7	4.0
Number of appointments until FP suspected CRC		
1	28	16.0
2	41	23.4
3	36	20.6
4	7	4.0
5	3	1.7
>5	5	2.9
Other	11	6.3
N/A	44	25.1
Diagnostic tests resulting in diagnosis		
Digital rectum exam	38	21.7
Colonoscopy	107	61.1
CT colonography	39	22.3
Flexible sigmoidoscopy	19	10.9
Blood tests	35	20.0
Biopsy	35	20.0
Computed tomography (CT) scan	24	13.7
Positron emission tomography (PET) scan	4	2.3
Magnetic resonance imaging (MRI) scan	16	9.1
Ultrasound scan of the rectum	12	6.9
Ultrasound scan of abdomen	1	0.6
Other	6	3.4
N/A	8	4.6
Paid for private testing to obtain CRC diagnosis		
Yes	55	31.4
No	107	61.1
Unsure	4	2.3
Other	1	0.6
N/A	8	4.6

**Table A9:** Patient experience post-CRC diagnosis.

Variable	Number of Participants (n)	Proportion (%)
Felt that delay experienced was due to feeling dismissed by an FP early-on in the process		
Yes	59	33.7
No	95	54.3
Other	11	6.3
N/A	10	5.7
Felt the need to self-advocate for symptoms to be taken seriously		
Yes	111	63.4
No	35	20.0
Did not experience symptoms	21	12.0
Other	3	1.7
N/A	5	2.9
Sought a second opinion after misdiagnosis		
Yes	52	29.7
No	52	29.7
Other	5	2.9
Not misdiagnosed	64	36.6
N/A	2	1.1
