# Supplementary material for: Understanding Colorectal Cancer Patient Experiences with Family Practitioners in Canada
Source: Curr Oncol. 2024 May 30;31(6):3122–48. doi: 10.3390/curroncol31060237 (PMC11202889; doi:10.3390/curroncol31060237)
Supplement: Supplementary file 1 [file curroncol-31-00237-s001.zip › curroncol-2970947-supplementary.pdf]

Supplemental Materials

1.0 Additional Details about Survey Questions

1.1. Demographic Variables

The selection of demographic variables was guided by those included in previous CCC studies and national datasets. Demographic variables included sex, age group, level of education, ethnicity/race, gender identity, and community type. Such characteristics were descriptively analyzed to provide an overview of the survey sample.

1.2. CRC Awareness

Four questions focused on the patient's awareness of CRC before their diagnosis. These questions addressed concepts such as knowledge of the symptoms and risk factors of CRC, early-age onset (EAO) CRC, and routine screening procedures. For each concept, respondents could select between 'Yes', 'Maybe', 'No', or 'Other' to describe their pre-diagnosis awareness. Proportions were calculated for each response option and for the grouped concepts to indicate the total awareness of each respondent. To further identify any underlying trends, responses were also stratified by age.

1.3. Pre-Diagnosis

The 'Pre-diagnosis' section contained thirty-four questions focused on patients' experience with family practitioners (FPs) before their CRC diagnosis. The *Symptoms* subsubsection contained dichotomous (i.e., yes/no), nominal (e.g., symptom type), and continuous (i.e., age) response data. The *Pre-Appointment* subsubsection contained nominal and ordinal (e.g., time waited) categorical responses. The *Appointment* subsubsection featured scale, nominal, and ordinal responses. The *Details of Experience with Family Practitioner* and *After Initial Appointment* subsubsections contained nominal and ordinal responses. Proportions were calculated for categorical responses, and central tendency measures were calculated for continuous responses. Age stratification was conducted to further identify any underlying trends. Moreover, ordinal variables from this section were compared with variables presented in the CRC awareness section to identify potential Spearman rho associations. The significance was set a priori at $p < 0.05$.

1.4. Eventual Diagnosis and Post-Diagnosis

The last section, 'Eventual Diagnosis and Post-Diagnosis', of fourteen questions focused on the eventual and post-diagnosis experiences of patients with their FPs. Response types featured in this section include continuous, ordinal, and nominal data. Quantitative methods were used to analyze the data from these responses. This section also included an open-ended question for patients to emphasize any additional points about their experience. The Braun & Clarke framework was used to extract key themes from these responses.

Supplementary Table S1: All Spearman's rho (ρ) associations tested using SPSS.

Variable A	Variable B	ρ	p Value (2-tailed)	95% CI (2-Tailed)	
				Lower	Upper
Did not feel that FP was dismissive of their CRC symptoms	Level of Education	0.035	0.645	-0.118	0.187
Somewhat felt that FP was dismissive of their CRC symptoms	Level of Education	-0.224	0.493	-0.101	0.203
Did feel that FP was dismissive of their CRC symptoms	Level of Education	0.052	0.232	-0.24	0.063
Did not feel that FP was dismissive of their CRC symptoms	Were not asked of their family history of CRC or polyps by their family physician	-0.139	0.067	-0.286	0.014
Somewhat felt that FP was dismissive of their CRC symptoms	Were not asked of their family history of CRC or polyps by their family physician	0.119	0.117	-0.034	0.267
Did feel that FP was dismissive of their CRC symptoms	Were not asked of their family history of CRC or polyps by their family physician	0.113	0.138	-0.041	0.261
Did not feel that FP was dismissive of their CRC symptoms	Were asked of their family history of CRC or polyps by their family physician	0.147	0.052	-0.006	0.293
Somewhat felt that FP was dismissive of their CRC symptoms	Were asked of their family history of CRC or polyps by their family physician	-0.097	0.203	-0.246	0.057
Did feel that FP was dismissive of their CRC symptoms	Were asked of their family history of CRC or polyps by their family physician	-0.041	0.597	-0.192	0.113
Did not feel that FP was dismissive of their CRC symptoms	Cancer stage at diagnosis	-0.139	0.08	-0.293	0.021
Somewhat felt that FP was dismissive of their CRC symptoms	Cancer stage at diagnosis	0.122	0.125	-0.39	0.277

Did feel that FP was dismissive of their CRC symptoms	Cancer stage at diagnosis	-0.005	0.953	-0.165	0.156
Did not feel that FP was dismissive of their CRC symptoms	Were not aware of CRC symptoms prior to diagnosis	-0.34	0.653	-0.186	0.119
Somewhat felt that FP was dismissive of their CRC symptoms	Were not aware of CRC symptoms prior to diagnosis	0.1	0.187	-0.053	0.249
Did feel that FP was dismissive of their CRC symptoms	Were not aware of CRC symptoms prior to diagnosis	-0.156	0.039	-0.302	-0.004
Did not feel that FP was dismissive of their CRC symptoms	Unsure of CRC symptom awareness prior to diagnosis	0.244	0.001	0.095	0.383
Somewhat felt that FP was dismissive of their CRC symptoms	Unsure of CRC symptom awareness prior to diagnosis	-0.033	0.661	-0.185	0.12
Did feel that FP was dismissive of their CRC symptoms	Unsure of CRC symptom awareness prior to diagnosis	-0.092	0.226	-0.241	0.062
Did not feel that FP was dismissive of their CRC symptoms	Were aware of CRC symptoms prior to diagnosis	-0.125	0.098	-0.273	0.028
Somewhat felt that FP was dismissive of their CRC symptoms	Were aware of CRC symptoms prior to diagnosis	-0.098	0.199	-0.247	0.056
Did feel that FP was dismissive of their CRC symptoms	Were aware of CRC symptoms prior to diagnosis	0.209	0.006	0.058	0.35
Did not feel that FP was dismissive of their CRC symptoms	Length of time to be diagnosed with CRC, after first seeking medical help	-0.038	0.631	-0.197	0.123
Somewhat felt that FP was dismissive of their CRC symptoms	Length of time to be diagnosed with CRC, after first seeking medical help	-0.125	0.117	-0.279	0.036
Did feel that FP was dismissive of their CRC symptoms	Length of time to be diagnosed with CRC, after first seeking medical help	-0.05	0.531	-0.209	0.111

Was not MISTAKENLY diagnosed with another condition prior to their CRC diagnosis	Level of Education	-0.08	0.291	-0.23	0.073
Was MISTAKENLY diagnosed with another condition prior to their CRC diagnosis	Level of Education	0.135	0.075	-0.018	0.282
Was not MISTAKENLY diagnosed with another condition prior to their CRC diagnosis	Were not asked of their family history of CRC or polyps by their family physician	-0.115	0.128	-0.263	0.038
Was MISTAKENLY diagnosed with another condition prior to their CRC diagnosis	Were not asked of their family history of CRC or polyps by their family physician	0.131	0.083	-0.022	0.278
Was not MISTAKENLY diagnosed with another condition prior to their CRC diagnosis	Were asked of their family history of CRC or polyps by their family physician	0.079	0.298	-0.074	0.229
Was MISTAKENLY diagnosed with another condition prior to their CRC diagnosis	Were asked of their family history of CRC or polyps by their family physician	-0.032	0.67	-0.184	0.121
Was not MISTAKENLY diagnosed with another condition prior to their CRC diagnosis	Cancer stage at diagnosis	-0.118	0.139	-0.273	0.043
Was MISTAKENLY diagnosed with another condition prior to their CRC diagnosis	Cancer stage at diagnosis	0.09	0.262	-0.072	0.246
Was not MISTAKENLY diagnosed with another condition prior to their CRC diagnosis	Were not aware of CRC symptoms prior to diagnosis	0.024	0.755	-0.129	0.176
Was MISTAKENLY diagnosed with another condition prior to their CRC diagnosis	Were not aware of CRC symptoms prior to diagnosis	-0.052	0.495	-0.203	0.102
Was not MISTAKENLY diagnosed with another condition prior to their CRC diagnosis	Unsure of CRC symptom awareness prior to diagnosis	0.131	0.084	-0.022	0.278

Was MISTAKENLY diagnosed with another condition prior to their CRC diagnosis	Unsure of CRC symptom awareness prior to diagnosis	-0.106	0.162	-0.255	0.047
Was not MISTAKENLY diagnosed with another condition prior to their CRC diagnosis	Were aware of CRC symptoms prior to diagnosis	-0.11	0.149	-0.258	0.044
Was MISTAKENLY diagnosed with another condition prior to their CRC diagnosis	Were aware of CRC symptoms prior to diagnosis	0.118	0.12	-0.035	0.266
Was not MISTAKENLY diagnosed with another condition prior to their CRC diagnosis	Length of time to be diagnosed with CRC, after first seeking medical help	0.041	0.604	-0.119	0.2
Was MISTAKENLY diagnosed with another condition prior to their CRC diagnosis	Length of time to be diagnosed with CRC, after first seeking medical help	-0.073	0.362	-0.23	0.088
Cancer stage at diagnosis	Length of time to be diagnosed with CRC, after first seeking medical help	0.06	0.463	-0.105	0.222
Were not aware of CRC symptoms prior to diagnosis	Were not asked of their family history of CRC or polyps by their family physician	0.101	0.182	-0.052	0.25
Unsure of CRC symptom awareness prior to diagnosis	Were not asked of their family history of CRC or polyps by their family physician	-0.174	0.021	-0.318	-0.022
Were aware of CRC symptoms prior to diagnosis	Were not asked of their family history of CRC or polyps by their family physician	-0.003	0.964	-0.156	0.149
Were not aware of CRC symptoms prior to diagnosis	Were asked of their family history of CRC or polyps by their family physician	-0.079	0.296	-0.299	0.074
Unsure of CRC symptom awareness prior to diagnosis	Were asked of their family history of CRC or polyps by their family physician	0.158	0.037	0.005	0.303

Were aware of CRC symptoms prior to diagnosis	Were asked of their family history of CRC or polyps by their family physician	0.011	0.889	-0.142	0.163
Were not aware of CRC symptoms prior to diagnosis	Cancer stage at diagnosis	0.211	0.008	0.052	0.359
Unsure of CRC symptom awareness prior to diagnosis	Cancer stage at diagnosis	-0.101	0.207	-0.257	0.061
Were aware of CRC symptoms prior to diagnosis	Cancer stage at diagnosis	-0.106	0.183	-0.262	0.055
Were not aware of CRC symptoms prior to diagnosis	Length of time to be diagnosed with CRC, after first seeking medical help	0.243	0.002	0.086	0.388
Unsure of CRC symptom awareness prior to diagnosis	Length of time to be diagnosed with CRC, after first seeking medical help	-0.091	0.252	-0.248	0.07
Were aware of CRC symptoms prior to diagnosis	Length of time to be diagnosed with CRC, after first seeking medical help	-0.172	0.03	-0.323	-0.012

2.0 Colorectal Cancer Patient Experiences with Family Practitioners in Canada Survey

Welcome to the Colorectal Cancer Patient Experiences with Family Practitioners* in Canada Survey! The objective of this study is to explore the primary experiences of Canadian colorectal cancer (CRC) patients with their family practitioners during their pre-diagnosis, eventual diagnosis, and post-diagnosis. You will be asked questions about your: demographics, awareness of CRC, and experiences before, during, and after your CRC diagnosis.

The study should take you around 15-20 minutes to complete. You will have the option of providing your email to be entered in a random draw to win one of ten $25 Amazon e-gift cards and/or receive information from Colorectal Cancer Canada about future surveys, newsletters, support groups, and other research opportunities. Your participation in this research is voluntary. You have the right to withdraw at any point during the study. The Student Investigator (Patil Mksyartinian) of this study can be contacted at pmksyart@uwaterloo.ca.

*In the context of this study, the term Family Practitioner is synonymous with family physician/doctor, primary care provider, and general practitioner (GP).

Please carefully read the information-consent sheet before beginning the study: Information consent sheet By clicking the button below, you acknowledge:

- Read the Information-Consent Sheet
- You are at least 18 years of age.
- Diagnosed with colorectal cancer (CRC) within the last 10 years.
- Living in Canada.
- Able to provide virtual consent.
- Willing and able to complete the survey in English or French.

Please note that this survey will be best displayed on a laptop or desktop computer. Some features may be less compatible for use on a mobile device.

☐ I consent, begin the study

☐ I do not consent, I do not wish to participate in this study (please close the survey now

CRC Patient Demographic Questions

This set of questions will focus on your demographics, such as your age, race, ethnicity, gender, education, and location.

1. How old are you now?
☐ 0-19
☐ 20-29
☐ 30-39
☐ 40-49
☐ 50-59
☐ 60+

2. Are you: (Check all that apply)
☐ Arab
☐ Black
☐ Chinese
☐ Filipino
☐ Japanese
☐ Korean
☐ Latin American
☐ First Nations, Métis, Inuit
☐ South Asian (e.g., East Indian, Pakistani, Sri Lankan)
☐ Southeast Asian (e.g., Vietnamese, Cambodian, Laotian, Thai)
☐ West Asian (Iranian, Afghan, etc.)
☐ White
☐ Other (Please specify: ______)
☐ Prefer not to answer

3. Which gender do you most identify with?
☐ Man/Male
☐ Women/Female
☐ Trans*/Non-binary
☐ Prefer to self-identify (Please specify: ______)
☐ Prefer to not answer

4. What is the highest degree or level of education you have completed?
☐ Less than high school diploma
☐ High school diploma or equivalent
☐ CÉGEP ☐ College certificate/diploma/trade college
☐ Bachelor's degree
☐ Postgraduate certificate/diploma
☐ Juris Doctor (JD) degree

☐ Master’s degree
☐ Doctorate
☐ Other (Please specify: _______)

5. In which part of Canada do you live?
☐ Alberta
☐ British Columbia
☐ Manitoba
☐ New Brunswick
☐ Newfoundland & Labrador
☐ Northwest Territories
☐ Nova Scotia
☐ Nunavut
☐ Ontario
☐ Prince Edward Island
☐ Quebec
☐ Saskatchewan
☐ Yukon

6. What best describes where you currently live?
☐ Urban
☐ Suburban
☐ Rural
☐ Other (Please Specify: _____)

CRC Patient Awareness Questions

This set of questions will focus on your awareness of colorectal cancer (CRC), such as your knowledge of the symptoms of CRC, risks factors, early-age onset (EAO) CRC, and routine screening procedures.

7. Prior to diagnosis, were you aware colorectal cancer can happen in people younger than 50 years of age?
☐ Yes
☐ Maybe
☐ No
☐ Other (please specify: __________________)

8. Did you know the symptoms of colorectal cancer BEFORE being diagnosed?
☐ Yes
☐ Maybe
☐ No
☐ Other (please specify: __________________)

9. Were you aware of the routine colorectal cancer screening procedures (e.g., FIT/Stool test, sigmoidoscopy, colonoscopy, etc.)?
☐ Yes
☐ Maybe
☐ No

☐ Other (please specify: __________________)

10. Have you ever discussed your family health history, including colorectal cancer risk factors, with your family or family practitioner BEFORE your diagnosis?
☐ Yes, but only with my family member(s)
☐ Yes, but only with my family practitioner
☐ Yes, with both my family member(s) and my family practitioner
☐ I did not discuss colorectal cancer risk factors with my family member(s) or family Practitioner

Patient Experience: Pre-Diagnosis Questions

This set of questions will focus on your pre-diagnosis experience with your family practitioner, which comprises your experiences before you were diagnosed with colorectal cancer (CRC). This includes your experiences with your symptoms and details of your experiences with your family practitioner before, during, and after your initial appointment.

Symptoms

11. Did you experience any colorectal cancer (CRC) symptoms before being diagnosed with CRC?
☐ Yes
☐ No (Skip to Question 15)

12. At what age did you first notice your symptoms?
____

13. Which of the following symptoms did you experience? (Check all that apply)
☐ Diarrhea
☐ Constipation
☐ Weight loss
☐ Blood in stool
☐ Rectal Bleeding
☐ "Unusual" fatigue
☐ Decreased appetite
☐ Persistent cramps
☐ Bowels never feel empty
☐ Bloating and/or gas
☐ Nausea and/or vomiting
☐ Abdominal pain
☐ Other (If other symptoms, please specify: __________________)

14. Did you suscept that these symptoms were related to colorectal cancer?
☐ Yes
☐ No
☐ Other (please specify: __________________)

Pre-Appointment

15. Did you have a family practitioner before being diagnosed with colorectal cancer (CRC)?

☐ Yes
☐ No
☐ Other (please specify: ________________)

16. How long did you experience symptoms before visiting a family practitioner?
☐ Less than 1 month
☐ 1-3 months
☐ 3-6 months
☐ 6-12 months
☐ Greater than 1 year
☐ Not applicable, no symptoms were experienced
☐ Other (please specify: ________________)

17. Did you delay getting an appointment with a family practitioner?
☐ Yes
☐ No (Skip to Question 19)
☐ Other (please specify: ________________)

18. What challenges delayed your ability to get an appointment? (Check all that apply)
☐ COVID-19
☐ Financial constraints
☐ Workplace/occupation
☐ Caregiver burden
☐ Travel time
☐ Rural location (distance)
☐ Lack of access to family practitioner
☐ Other (please specify: ________________)

19. How long did it take to get your appointment with a family practitioner?
☐ Less than 1 week
☐ 1-2 weeks
☐ 3 weeks
☐ 4-6 weeks
☐ Greater than 6 weeks
☐ Other (please specify: ________________)

20. Did your symptoms worsen while waiting for an appointment?
☐ Yes
☐ No
☐ Not applicable, no symptoms were experienced
☐ Not applicable, I did not need to wait for an appointment (Skip to Question 22)

21. Did you seek emergency care while waiting for your appointment?
☐ Yes (explain if needed: ________________)
☐ No

22. Did a routine screening procedure (e.g., a FIT test) lead you to seek an appointment with a family practitioner?
☐ Yes (please specify: ___________________)
☐ No
☐ Other (please specify: ___________________

Appointment

23. At what age did you visit a family practitioner to discuss colorectal cancer?
____

24. Did anyone come with you during appointment with a family practitioner?
☐ Partner/spouse
☐ Friend
☐ Sibling
☐ Child(ren)
☐ Went alone
☐ Other (please specify: ___________________)

25. What were you stress levels during your initial appointment? (0-meaning no stress and 10-meaning a very high level of stress)
(A scale rating tool was used)

26. Was your initial appointment with a family practitioner virtual (e.g., a phone call)?
☐ Yes
☐ No
☐ Other (please specify: ___________________)

Details of Experience with Family Practitioner

27. Did you ask your family practitioner if your symptoms were related to colorectal cancer?
☐ Yes
☐ No
☐ Not applicable, no symptoms were experienced
☐ Other (please specify: ___________________)

28. Did you feel your family practitioner was concerned about your symptoms and/or screening results?
☐ Yes
☐ No
☐ Other (please specify: ___________________)

29. Did you feel your family practitioner dismissed your colorectal cancer symptoms?
☐ Yes
☐ No (Skip to Question 31)
☐ Somewhat
☐ Not applicable, no symptoms were experienced

☐ Other (please specify: __________________)

30. If you did feel dismissed, do you think it was due to your age?
☐ Yes
☐ No
☐ Other (please specify: __________________)

31. Did you feel you had to advocate for your diagnosis to be taken seriously?
☐ Yes
☐ No
☐ Not applicable, no symptoms were experienced
☐ Other (please specify: __________________)

32. Were you MISTAKENLY diagnosed with any of these conditions prior to your diagnosis of colorectal cancer? (Check all that apply)
☐Hemorrhoids
☐ Symptoms of childbirth
☐ Appendicitis
☐ Gynecological issues
☐ Irritable Bowel Syndrome (IBS)
☐ Irritable Bowel Disease (IBD)
☐ Mental health issues
☐ Crohn's/Colitis
☐ I was not misdiagnosed (Skip to Question 35)
☐ Other (please specify: __________________)

33. Did you dismiss your colorectal cancer symptoms because you were initially misdiagnosed?
☐ Yes
☐ No
☐ Other (please specify: __________________)

34. Did you seek a second opinion after you were misdiagnosed?
☐ Yes
☐ No
☐ Other (please specify: __________________)

35. According to you, what was the possible reason that your symptoms were not fully considered for colorectal cancer? (Check all that apply)
☐ I was young when I first had my symptoms
☐ My symptoms were relatively mild
☐ I did not have a family history of colorectal cancer
☐ I had been affected by one or more of the above selected conditions (e.g., Crohn’s)
☐ Not applicable, my symptoms were rightly diagnosed in time
☐ Not applicable, I did not experience any symptoms before being diagnosed with CRC
☐ Other (please specify: __________________)

36. What specialist(s) did your family practitioner refer you to? (Check all that apply)

☐ Emergency Room (ER) Doctor
☐ Gastroenterologist
☐ Oncologist
☐ Obstetrician-Gynecologist (OBGYN)
☐ Urologist
☐ Pediatrician
☐ Surgeon
☐ Not applicable, I was not referred by a family practitioner (Skip to Question 38)
☐ Other (please specify: __________________)

37. How long did it take before you were referred to a specialist by your family practitioner?
☐ Less than 1 month
☐ 1-3 months
☐ 3-6 months
☐ 6-12 months
☐ Greater than 1 year
☐ Other (please specify: __________________)

38. Did your family practitioner ask if you had family history of colorectal cancer or polyps?
☐ Yes
☐ No
☐ Other (please specify: __________________)

39. Did your family practitioner order genetic testing for colorectal cancer?
☐ Yes
☐ No
☐ Not applicable, genetic testing was not needed
☐ Not applicable, other health care provider ordered genetic testing (please specify: __________________)

40. Which test(s) did your family practitioner order for screening for colorectal cancer? (Check all that apply)

☐ Fecal Immunochemical Test (FIT)/ Fecal Occult Blood Test (FOBT)/Immunochemical fecal occult test (IFOBT)
☐ Colonoscopy
☐ Digital rectum exam
☐ CT Colonography
☐ Flexible sigmoidoscopy
☐ Blood tests
☐ Biopsy
☐ Not applicable, other health care provider ordered screening test(s) (please specify: __________________)
☐ Other (please specify: __________________)

41. How long did it take to get your screening test completed?

☐ 1-2 weeks
☐ Less than 1 month
☐ 1-3 months
☐ 3-6 months
☐ 6-12 months
☐ Greater than 1 year
☐ Other (please specify: ___________________)

After Initial Appointment

42. How long did it take to get your test results back from your family practitioner?
☐ Less than 1 week
☐ 1-2 weeks
☐ 3 weeks
☐ 4-6 weeks
☐ Greater than 6 weeks
☐ Not applicable, other health care provider provided test results (please specify: ___________________)
☐ Other (please specify: ___________________)

43. Did your family practitioner discuss your test results with you?
☐ Yes
☐ No
☐ Not applicable, other health care provider discussed results (please specify: ___________________)
☐ Unsure

44. How long did it take until your family practitioner suspected colorectal cancer (CRC), after you first sought medical help?
☐ Less than 1 month
☐ 1-3 months
☐ 3-6 months
☐ 6-12 months
☐ Greater than 1 year
☐ Not applicable, other health care provider suspected CRC (Skip to Question 46) (please specify: ___________________)
☐ Other (please specify: ___________________)

45. How many appointments did you have until your family practitioner suspected colorectal cancer?
☐ 1
☐ 2
☐ 3
☐ 4
☐ 5
☐ More than 5 appointments
☐ Other (please specify: ___________________)

Patient Experience: Eventual Diagnosis and Post-Diagnosis Questions

This set of questions will focus on your eventual diagnosis and post-diagnosis experiences with your family practitioner, which comprises your experiences during your colorectal cancer (CRC) diagnosis and after your CRC diagnosis.

46. At what age were your diagnosed with colorectal cancer?
____

47. What is your date of diagnosis? (DD/MM/YYYY) or (MM/YYYY)
____

48. Did you have to pay for private testing to get diagnosed with colorectal cancer?
☐ Yes
☐ No
☐ Unsure
☐ Other (please specify: __________________)

49. Did you feel you experienced a delay in your colorectal cancer diagnosis because you felt dismissed by your family practitioner early-on?
☐ Yes
☐ No
☐ Other (please specify: __________________)

50. Which diagnostic test resulted in your colorectal cancer diagnosis? (Check all that apply)
☐ Digital rectum exam
☐ CT Colonography
☐ Flexible sigmoidoscopy
☐ Blood tests
☐ Biopsy
☐ Computed Tomography (CT) scan
☐ Positron Emission Tomography (PET) scan
☐ Magnetic Resonance Imaging (MRI) scan
☐ Ultrasound scan of the rectum
☐ Ultrasound scan of abdomen
☐ Other (please specify: __________________)

51. Did your family practitioner recommended anything to help manage your colorectal cancer? (Check all that apply)
☐ Pharmaceutical (e.g., medications)
☐ Resources (e.g., educational materials)
☐ Referral to social worker
☐ Support groups
☐ Not applicable, my family practitioner did not recommend anything
☐ Other (please specify: __________________)

52. Which health care professional diagnosed you with colorectal cancer?
☐ Family Practitioner

☐ Emergency Room (ER) Doctor
☐ Gastroenterologist
☐ Oncologist
☐ Obstetrician-Gynecologist (OBGYN)
☐ Urologist
☐ Pediatrician
☐ Surgeon
☐ Other (please specify: ___________________)

53. How long did it take to be diagnosed with colorectal cancer, after you first sought medical help?
☐ Less than 1 month
☐ 1-3 months
☐ 3-6 months
☐ 6-12 months
☐ Greater than 1 year
☐ Other (please specify: ___________________)

54. What type of cancer were you diagnosed with?
☐ Colon
☐ Rectal
☐ Unsure
☐ Other (please specify: ___________________)

55. What stage of colorectal cancer were you diagnosed with?
☐ Stage 0
☐ Stage 1 (I)
☐ Stage 2 (II)
☐ Stage 3 (III)
☐ Stage 4 (IV)
☐ Unsure
☐ Other (please specify: ___________________)

56. Did you understand and feel fully informed about your diagnosis when it was explained to you by your family practitioner?
☐ Yes
☐ No
☐ My diagnosis was explained by a different health care provider (Skip to Question 59)
☐ Other (please specify: ___________________)

57. Did your family practitioner use plain language to discuss your diagnosis?
☐ Yes
☐ No
☐ Other (please specify: ___________________)

58. How did your family practitioner explain your colorectal cancer diagnosis? (Check all that apply)
☐ Verbal explanation

☐ Visuals (e.g., diagrams/photos)
☐ Resources (pamphlets, booklets)
☐ Referral to Colorectal Cancer Canada website
☐ Other (please specify: __________________)

59. Do you have anything else you would like to add about your experience with your family practitioner in diagnosing your colorectal cancer?
____

Thank you Message and Prize Draw/Information Colorectal Cancer Canada
Thank you! For participating in the Colorectal Cancer Patient Experiences with Family Practitioners in Canada Survey.

Your responses are greatly appreciated. If you are interested in viewing the results of this survey, updates will be posted on Colorectal Canada Canada's website (https://www.colorectalcancercanada.com/).

Please make sure to click the arrow button below to save your responses, and to see details about a prize draw and information from Colorectal Cancer Canada.

This study has been reviewed and received ethics clearance through a University of Waterloo Research Ethics Board (REB #44584). If you have questions for the Board contact the Office of Research Ethics, at 1-519-888-4567 ext. 36005 or reb@uwaterloo.ca.

For all other questions or if you have general comments or questions related to this study, please contact Patil Mksyartinian at 905-435-6355 or pmksyart@uwaterloo.ca

End of Survey Message

We thank you for your time spent taking this survey. Your response has been recorded.
If you are interested in being entered in a draw for 1 of 10, $25 Amazon e-gift cards and/or receive information from Colorectal Cancer Canada regarding future research opportunities, newsletters, and more, please click this link.
